# Supplementary material for: Digit-tracking as a new tactile interface for visual perception analysis
Source: Nat Commun. 2019 Nov 26;10:5392. doi: 10.1038/s41467-019-13285-0 (PMC6879631; doi:10.1038/s41467-019-13285-0)
Supplement: Supplementary file 1 — Supplementary Information [file 41467_2019_13285_MOESM1_ESM.pdf]

Supplementary Information

Digit-tracking as a new tactile interface for visual perception analysis

Guillaume Lio[1,2,3], Roberta Fadda[4,5], Giuseppe Doneddu[5], Jean-René Duhamel[1,2*], Angela Sirigu[1,2*]

[1]Institute of Cognitive Science Marc Jeannerod, CNRS, Bron, France

[2]University of Lyon, France

[3] Reference Center for Rare Diseases with psychiatric phenotype Génopsy, le Vinatier Hospital, Bron, France.

[4]Azienda Ospedaliera Brotzu, Cagliari, Italy

[5]Department of Pedagogy, Psychology, Philosophy, University of Cagliari, Italy.

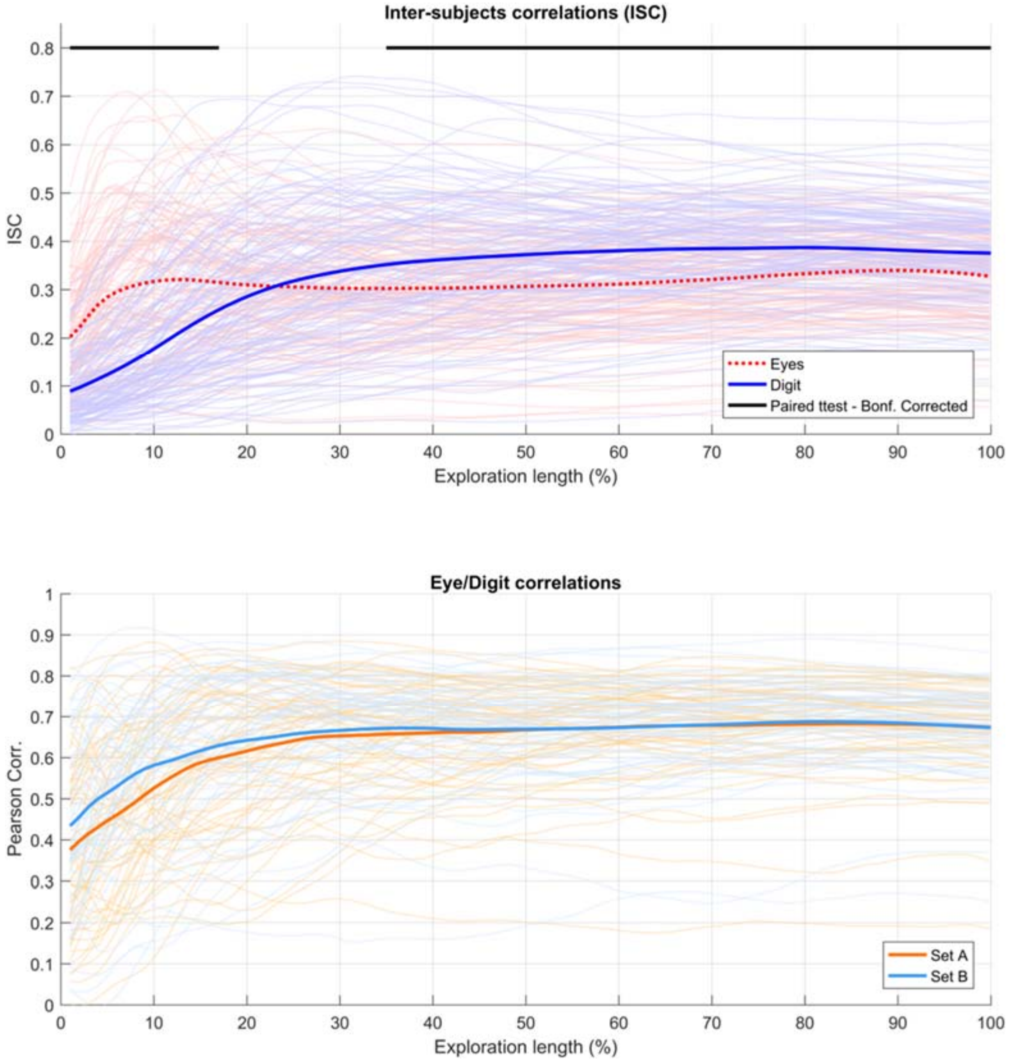

Supplementary Figure 1. Effects of exploration length on saliency map consistency. In order to determine how attention maps evolve over time and examine the impact on exploration statistics of a potential initial central bias due to the presence of a fixation cross (eye-tracking) or start button at the screen center, we took into account a third dimension of exploration length expressed as % total exploration time for eye-tracking and as % of total exploration distance for digit-tracking. In order to limit the computational load, eye-tracking and digit-tracking measurements were transposed in a 3D space of 100 x X pixels in space, where X= max(Height, Length)/min(Height,Length)*100) x 100 data points for exploration length (i.e. we decreased the exploration map resolution, while respecting width to height ratio of each image). These attention maps were processed using a 3d smoothing Gaussian kernel with a σ=3 in space and a σ=1 in time. The cumulative sum of the maps was then computed along the time dimension and normalized for each subject and at each time point by dividing the estimated densities by the maximum value. This procedure allowed to estimate the build-up of exploration maps along the exploration length. A) Cumulative inter-subject correlations (ordinate) were computed for each picture, with digit-tracking or eye-tracking, from 1% to 100% of exploration length (abscissa). Inter-subject correlations significantly higher for eye-tracking than digit-tracking in the early stages of exploration (1 to 17% of exploration length). This suggests that initial ISCs are inflated by a central location bias which, however, is not observed in the digit-tracking data. However, this bias vanishes as data accumulate (exploration length > 34%, ≈1.5s of eye-tracking data) and has a negligible impact on the final ISC estimates. B) Correlation between Eyes and Digit derived attention maps, from 1% to 100% of exploration. The correlations between eye- and digit-tracking attention maps are lowest below 20% of exploration length, most likely due to fact that the data obtained by the two methods are not biased in the same way at the early stages of exploration. However, this initial difference also has little impact on the final estimate of correlation between eye- and digit-tracking. Thin lines in both plots show cumulative values of individual images and the thick line represents the group average.

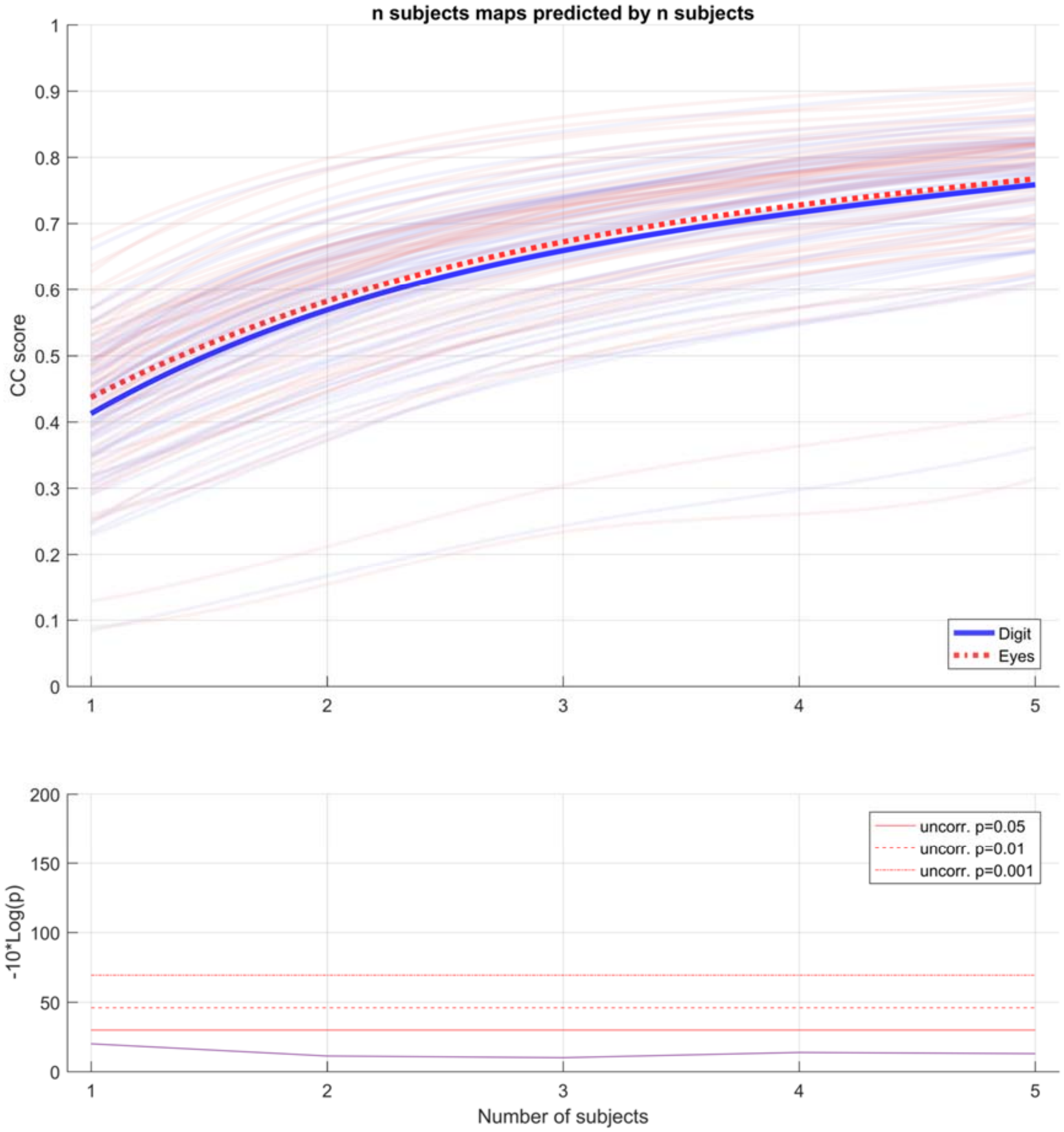

Supplementary Figure 2: Convergence analysis according to the method of Judd et al. (2012)[20] Each curve represents, for each picture, how much an attention map derived from N subjects correlates with another attention map derived from N other subjects. The figures on the left represent this analysis for digit-tracking compared with the standard eye-tracking method. No differences between the two recording strategies were found using this analysis (Uncorrected sign test p-values all >0.05).

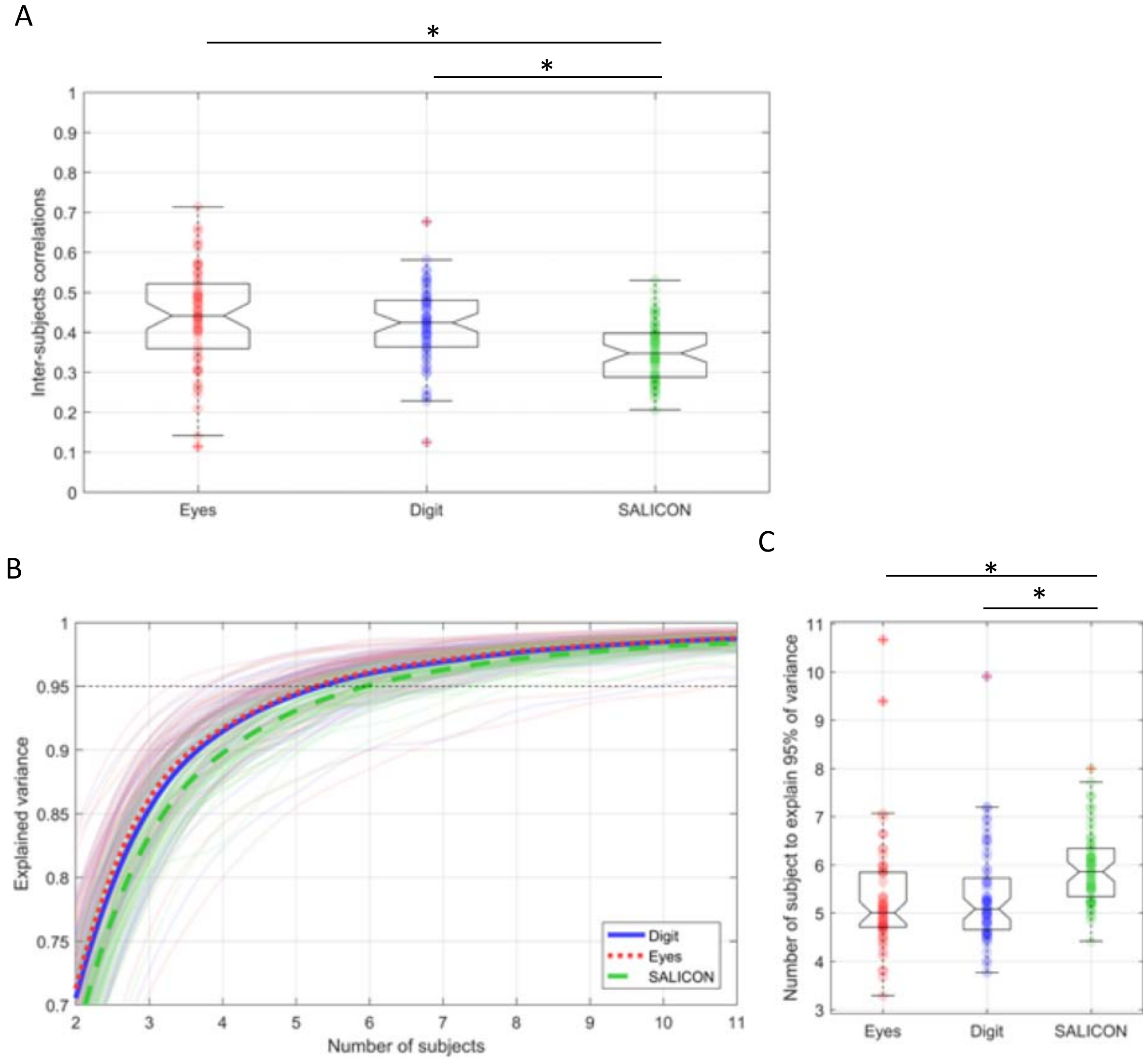

Supplementary Figure 3. Eye-, digit- and mouse-tracking comparison. A total of 61 pictures featuring humans, animals, objects or abstract art were used (Set A). Mouse-tracking was implemented using the same image degradation parameters as for digit-tracking but the method implementation was otherwise identical to Jiang et al.'s (2015). **A)** Plots of the Pearson's correlation coefficients calculated, for each picture, between the probability density estimates of exploration measured with eye-tracking, digit-tracking or mouse-tracking (11 subjects/condition). The first two techniques can measure precise attention maps that are highly correlated between subjects, but mouse-tracking shows significantly lower inter-subject correlations than eye-tracking ($p<5.4x10^{-4}$ sign test uncorrected) or digit-tracking ($p<4.9x10^{-5}$ sign test uncorrected). **B)** Convergence of exploration density estimates. Each curve represents for one image/technology couple recorded with N subjects, the percent of variance of the exploration density estimates that could be explained with N-1 subject. **C)** Plots of the number of subjects necessary to explain more than 95% of variance. Eye-tracking and digit-tracking show similar performances and require fewer subject than mouse-tracking to obtain stable measurements (eye v. mouse: $p<1.9x10^{-5}$ sign test uncorrected; digit v. mouse: $p<6.5x10^{-5}$ sign test uncorrected). These differences between the performance of the digit- and mouse-tracking implementations could reflect the objectives pursued by the two approaches. The mouse-tracking technique of Jiang et al (2015) was aimed mainly at identifying universally salient image features in large image data bases and does so relatively well by averaging out measurement noise across many subjects. In contrast, our digit-tracking was developed and optimized to achieve single-subject measurements that are as noise-free and reliable as possible (i.e. equivalent or better than eye-tracking) so that it could be used not only in group studies but also for individual diagnostic purposes.

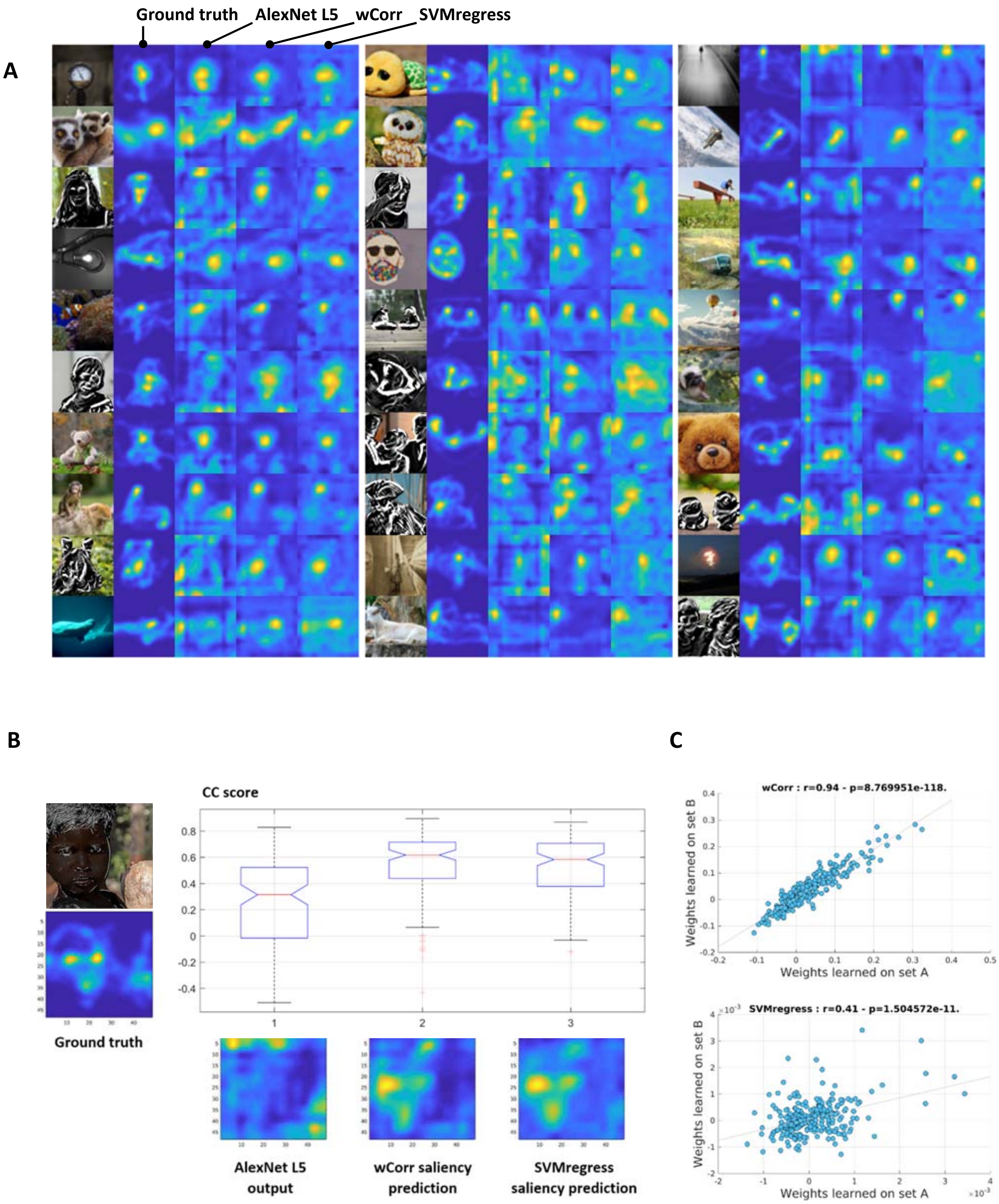

Supplementary Figure 4. A) Examples of saliency map generated with the CNN architecture for digit-tracking saliency exploration. The first column represents the input image, the second the measured saliency map. The third column represent the predicted saliency map calculated with a uniform weighting function and, the fourth and the fifth columns the predicted saliency maps with weights estimated with the wCorr and the SVMregress method respectively. **B)** Saliency map prediction quality, estimated using the Pearson Correlation Coefficient (CC score) between the predicted and the measured saliency map. Without any optimization, and as a benchmark

performance, the pretrained AlexNet Network can predict decently the saliency map on some of the images (All weights = 1. CC score = 0.31 $p<1x10^{-7}$ sign test – FWER corrected). The wCorr and the SVMregress methods obtain a prediction performance of 0.63 ($p<1x10^{-20}$ sign test – FWER corrected) and 0.63 ($p<1x10^{-23}$ sign test – FWER corrected) respectively. No significant differences have been found between the two learning strategy ($p>0.05$ sign test). **C)** The stability of the learned weights has been tested using a correlation analysis. For the wCorr and the SVMregress methods, the weighting function has been estimated on two different subsets of 61 images (set A and B), with saliency maps measured on two different populations of 10 subjects. A very strong correlation is observed between the weights learned using the wCorr method ($r_{pearson}=0.94$, $p<1x10^{-117}$) while the correlation observed for the SVMregress is far less obvious ($r_{pearson}=0.41$, $p<1x10^{-10}$). This result indicates a better robustness for the weight estimated with the wCorr method on small image datasets. This result suggests that the wCorr method might be a more biologically plausible approach for saliency-map prediction and that this learning strategy should be more appropriate to build saliency prediction at the single subject level (when a large amount of explored image cannot be recorded). Therefore, wCorr was the learning method privileged for the rest of the study. Please note that original human faces have been modified to hide individuals' identity.

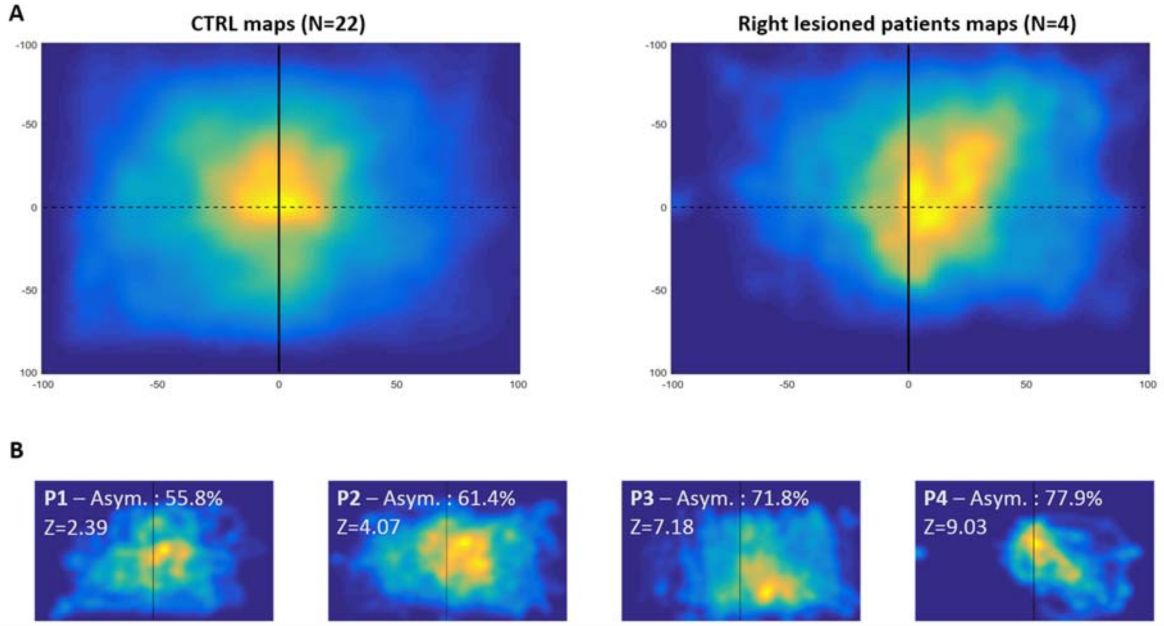

Supplementary Figure 5. Representative examples of the mean density of exploration obtained from patients with right hemisphere ischemic or hemorrhagic strokes. A) Left: Mean spatial distribution of digit-tracking explorations for a population of 22 control subjects. **Right:** Mean spatial distribution of digit-tracking explorations for a population of 4 patients, with right ischemic or hemorrhagic lesions. Note the tendency to neglect the left side of the pictures. **B**) Individual maps for each of these 4 patients. **P1**: Right ischemic stroke – Asymmetry right = 55.8%, Z=2.39. **P2**: Right ischemic stroke (Middle cerebral artery) – Asymmetry right = 61.4%, Z=4.07. **P3**: Right ischemic stroke (Middle cerebral artery – total) – Asymmetry right = 71.8%, Z=7.18. **P4**: Right hemorrhagic stroke – Asymmetry right = 77.9%, Z=9.03.

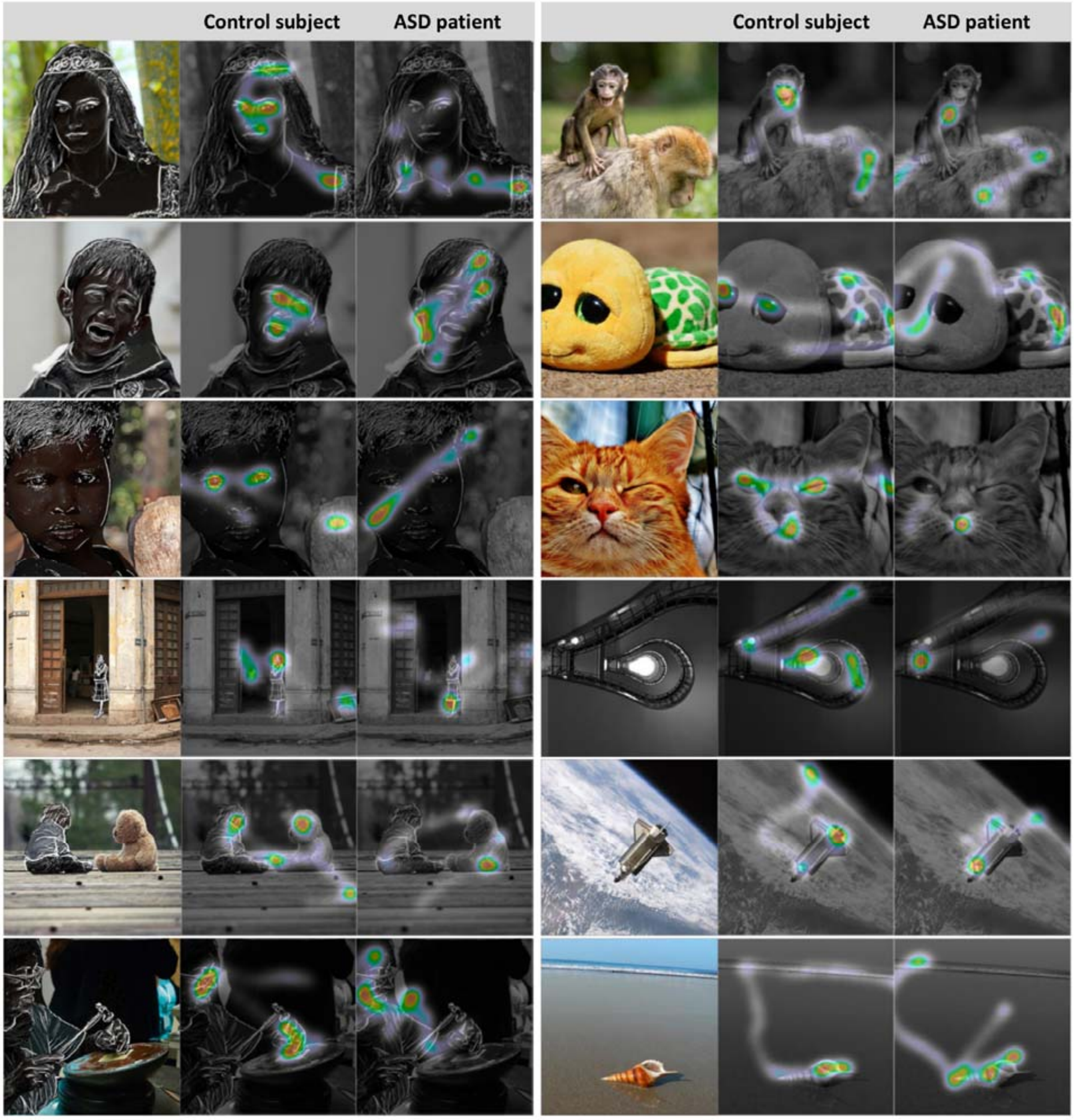

Supplementary Figure 6. Representative examples digit-tracking heat maps from a neurotypical subject and an autistic (ASD) patient. In contrast to the control subject **(**CTRL), ASD patient bypasses or underexplores the eye region in pictures containing large faces, or the face regions in those depicting social interactions. Note further the avoidance of an eye-like element in the picture of a spiral staircase and the absence of gaze following to attention focus of the human holding a hammer. Exploration of images containing only inanimate objects shows that ASD patients, like neurotypical subjects, focus on the most informative regions. Please note that original human faces have been modified to hide individuals' identity.

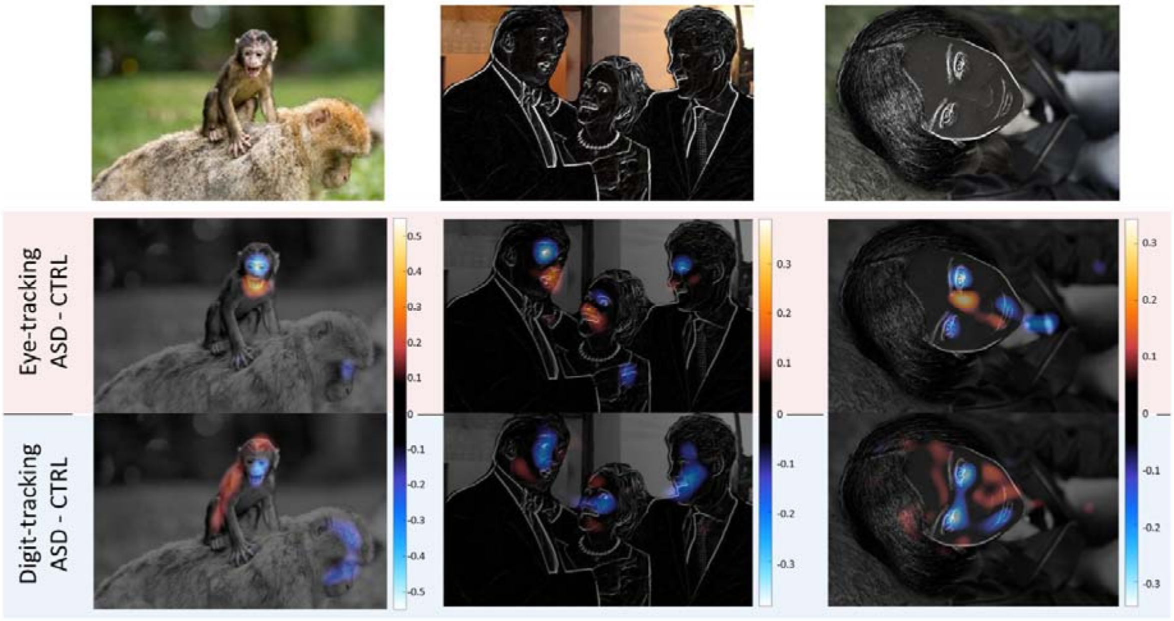

Supplementary Figure 7. Representative examples of contrasts maps obtained from a group of patients with autistic spectrum disorder (ASD) and a matched control group of neurotypical subjects (CTRL). The regions most observed by patients are represented with hot colors and regions most observed by the neurotypical population are represented with cold colors. Both methods reveal that ASD patients underexplore the eye region and focus more on less socially salient details like the contour of the face, hair or body. Please note that original human faces have been modified to hide individuals' identity.

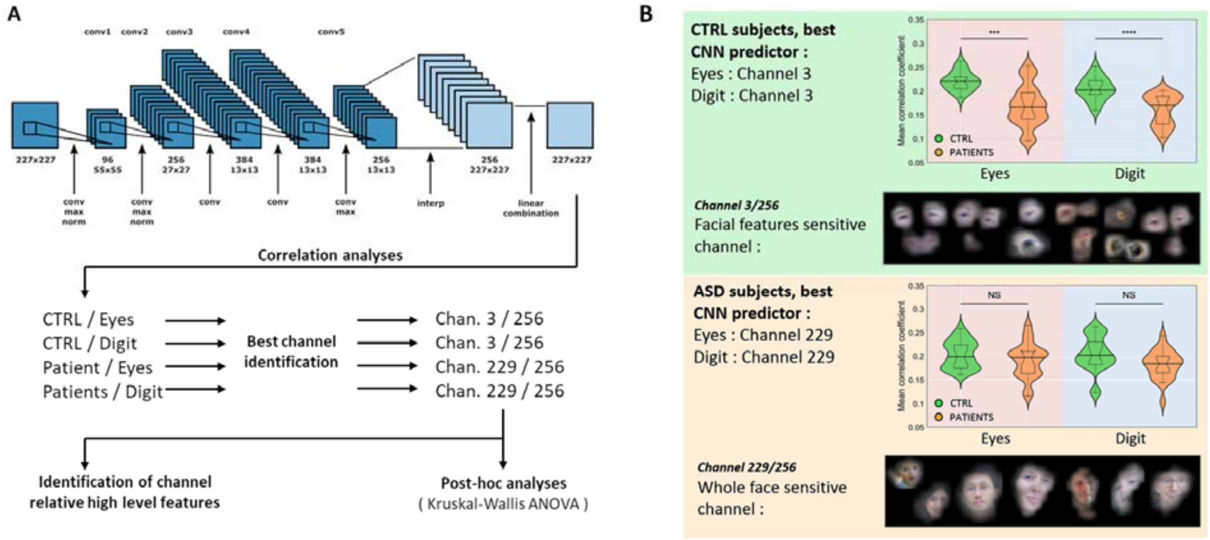

Supplementary Figure 8. Identification of the most salient features in attention maps obtained by eye and digit-tracking and for neurotypical and ASD populations using the AlexNet convolutional neural network. For each subject, 256 correlation scores were computed. Each score represents the mean correlation between a subject's attention map and the 256 CNN derived feature-maps (each CNN feature-map/channel represents a different high-level feature optimized for image classification). **A)** CNN architecture and methodological flowchart for the identification of the most salient high-level features in the neurotypical and ASD populations. The CNN channel that produces the best prediction of the measured heat-maps was identified with correlation analysis. Then, for each channel selected, a non-parametric post-hoc analysis was performed to identify if the measured correlation scores are significantly higher for one population than the other. Finally, the high-level features are visualized by identifying in the picture database the most responsive pixels for the considered CNN channel. **B)** With either measurement technique, the most salient high-level feature for the neurotypical population was CNN channel 3 ($p<0.004$ for both eye and digit-tracking). Post-hoc analysis reveals that correlation scores for this channel are significantly higher for the neurotypical than for the ASD population ($p_{eye}<0.001$, $p_{digit}<0.0001$, non-parametric rank test, FWER-corrected). This channel is particularly sensitive to internal facial features and especially eyes, consistent with previous observations that ASD patients underexplore facial social-cues. **C)** With either measurement technique, the most salient high-level feature for the ASD population was CNN channel 229 ($p<0.004$ for both eye and digit-tracking). Post-hoc analysis failed to reveal any differences in correlation scores for this channel between the two populations or measurement devices (Kruskal-Wallis ANOVA – Chi2=6.31 p=0.097). This channel is particularly sensitive to large regions containing faces. Together, these results indicate than even if ASD patients identify the most salient features of a scene and can orient their attention to faces, there is in this population a conspicuous deficit in the exploration of significant local facial cues. Post-hoc analyses failed to reveal differences in the attractiveness of the faces between our two tested populations (Kruskal-Wallis ANOVA – Chi2=6.31 p=0.097).

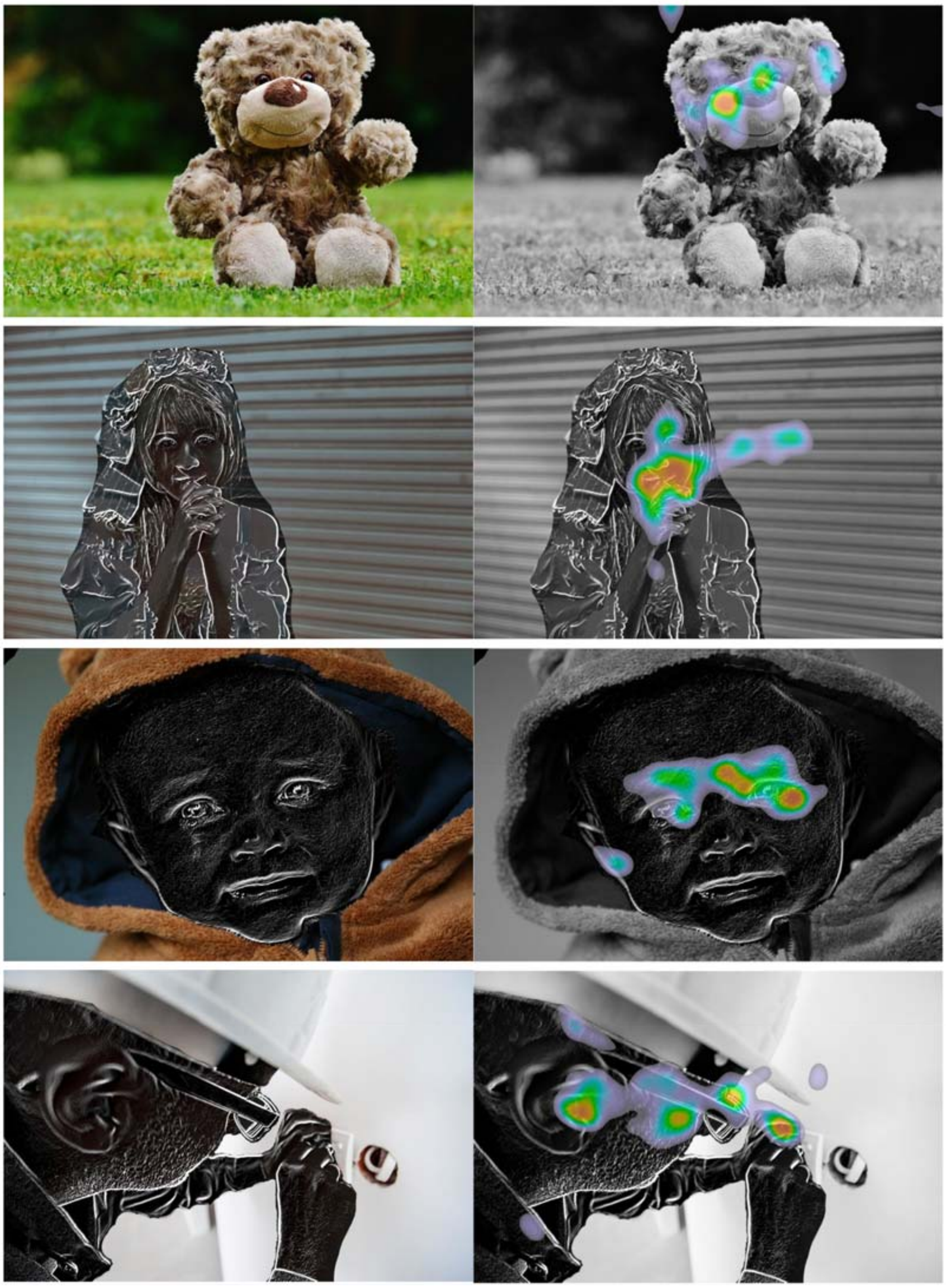

Supplementary Figure 9. Heat map examples obtained from a group of young children (N=3, mean age 3.0 years – σ=0.3) exploring images using the digit-track interface. Heat map overlay correspond to the mean attention map of the three subjects. The children were recruited and tested as part of an extended version of the adult protocol. All children were familiar with the display media but had only used it to watch videos and none had previous experience interacting with a tactile interface. A given image remained displayed until the subject had explored a fixed number of pixels equivalent to the height + the width of the screen size or when a time limit of 10s was reached. A short audio-visual animation serving as positive reinforcement was played upon completion of each image exploration. The recorded data was processed offline and analyzed in the same way as the human exploration data. Please note that original human faces have been modified to hide individuals' identity.

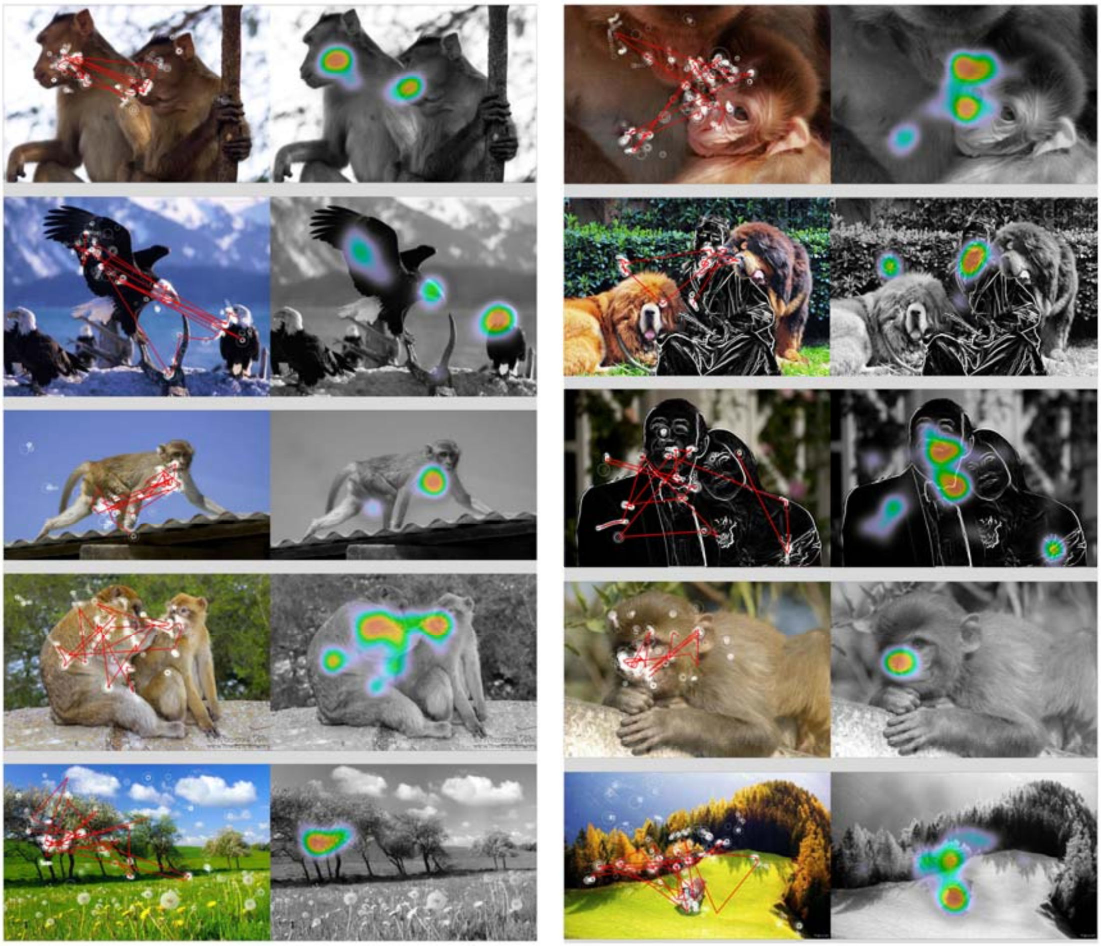

Supplementary Figure 10. Representative examples of scan paths and heat maps obtained from a male macaque monkey trained to use the digit-track interface. The finger trajectories of the monkey and heat maps are shown on the left and right version of each image, respectively. The monkey exhibits spontaneous attention to salient image features. Note, for example, the attraction to the two faces and the mother's nipple in the mother/infant picture (first row, right), and to the face and perineum regions of the female monkey (third row, left). **Methods.** *Subject:* One male monkey (*macaca fascicularis*, 6 year-old) living in a large enclosure with 3 other animals participated in this study. The monkey had free access to food and drink and was tested during a two-month period while no other experiment was taking place. *Procedure:* A remotely-controlled touch-sensitive display (768x1024 pixels) connected to an automatic food-delivering device was located above a platform inside the monkey's home cage. The animal was previously trained to touch images and obtain extra rewards (flavored sugar-free pellets not part of the animal's regular diet) for it. The digit-tracking software used in the human study served to present a set of 58 images. No time limit imposed and the monkey was free to approach the monitor, explore the displayed image with its finger and interrupt exploration at will. A given image remained displayed until the subject had explored a fixed number of pixels (During the training phase, the required distance of exploration was fixed to 50pixels for the first picture, 100 for the second, etc. to reach a maximum exploration length of 2000 pixels), after which a reward was automatically delivered and the next image presented. The recorded data was processed offline and analyzed in the same way as the human exploration data. Please note that original human faces have been modified to hide individuals' identity.
