# Supplementary material for: Digit-tracking as a new tactile interface for visual perception analysis
Source: Nat Commun. 2019 Nov 26;10:5392. doi: 10.1038/s41467-019-13285-0 (PMC6879631; doi:10.1038/s41467-019-13285-0)
Supplement: Supplementary file 3 — Reporting Summary [file 41467_2019_13285_MOESM3_ESM.pdf]

nature research

Corresponding author(s): Angela Sirigu

Last updated by author(s): 2019/10/29

Reporting Summary

Nature Research wishes to improve the reproducibility of the work that we publish. This form provides structure for consistency and transparency in reporting. For further information on Nature Research policies, see Authors & Referees and the Editorial Policy Checklist.

Statistics

For all statistical analyses, confirm that the following items are present in the figure legend, table legend, main text, or Methods section.

n/a	Confirmed	
☐	☒	The exact sample size (*n*) for each experimental group/condition, given as a discrete number and unit of measurement
☐	☒	A statement on whether measurements were taken from distinct samples or whether the same sample was measured repeatedly
☐	☒	The statistical test(s) used AND whether they are one- or two-sided<br>*Only common tests should be described solely by name; describe more complex techniques in the Methods section.*
☐	☒	A description of all covariates tested
☐	☒	A description of any assumptions or corrections, such as tests of normality and adjustment for multiple comparisons
☐	☒	A full description of the statistical parameters including central tendency (e.g. means) or other basic estimates (e.g. regression coefficient) AND variation (e.g. standard deviation) or associated estimates of uncertainty (e.g. confidence intervals)
☐	☒	For null hypothesis testing, the test statistic (e.g. *F*, *t*, *r*) with confidence intervals, effect sizes, degrees of freedom and *P* value noted<br>*Give P values as exact values whenever suitable.*
☒	☐	For Bayesian analysis, information on the choice of priors and Markov chain Monte Carlo settings
☐	☒	For hierarchical and complex designs, identification of the appropriate level for tests and full reporting of outcomes
☐	☒	Estimates of effect sizes (e.g. Cohen's *d*, Pearson's *r*), indicating how they were calculated

Our web collection on statistics for biologists contains articles on many of the points above.

Software and code

Policy information about availability of computer code

Data collection	Matlab (r2016a – the MathWorks, Inc.) custom code, designed using the Psychophysics Toolbox Version 3.<br>Clearview 2.7.0 - Tobii Technology
Data analysis	Matlab (r2016a to r2019a – the MathWorks, Inc.) custom code.

For manuscripts utilizing custom algorithms or software that are central to the research but not yet described in published literature, software must be made available to editors/reviewers. We strongly encourage code deposition in a community repository (e.g. GitHub). See the Nature Research guidelines for submitting code & software for further information.

Data

Policy information about availability of data

All manuscripts must include a data availability statement. This statement should provide the following information, where applicable:

- Accession codes, unique identifiers, or web links for publicly available datasets
- A list of figures that have associated raw data
- A description of any restrictions on data availability

Data on healthy subjects that supports the findings of this study and Matlab scripts are available on Open Science Framework http://osf.io/x5ryp . Patients' data are available from the corresponding authors on reasonable request.

Field-specific reporting

Please select the one below that is the best fit for your research. If you are not sure, read the appropriate sections before making your selection.

☐ Life sciences ☒ Behavioural & social sciences ☐ Ecological, evolutionary & environmental sciences

For a reference copy of the document with all sections, see nature.com/documents/nr-reporting-summary-flat.pdf

Behavioural & social sciences study design

All studies must disclose on these points even when the disclosure is negative.

Study description	Quantitative experimental study
Research sample	Two groups of participants were recruited for this study. One group of 22 subjects with no history of psychiatric or neurological disease (referred to as neurotypical or control (CTRL) group) served in the comparative evaluation of digit-tracking and eye-tracking technologies and as sex- and age-matched controls for ASD patients. The ASD group was composed of 22 male patients (mean age: 20, σ: 2.5) recruited at the Azienda Ospedaleria Brotzu (Cagliari, Italy) and with a clinical diagnosis of autism according to the Diagnostic and Statistical Manual of Mental Disorders, 5th edition. They were all rehabilitated patients with a large range of intelligence estimates (WAIS: range [58 - 141], mean: 96, σ: 22 (IVth edition - 2008)) and autistic symptoms intensity (ADOS45 – Mod IV : range [2 - 14], mean: 8.2 , σ: 3.4) at the time of the study. All participants had normal or corrected to normal vision.
Sampling strategy	The experiment consisted in recording picture explorations from the two populations using two recording devices: a standard infrared video-based eye-tracker and digit-tracking (both are described in the methods section of the paper).<br><br>A pre-determined sample size was determined based on the standard sample sizes published in eye-tracking studies and convergence analyses were included in the study to estimates the optimal sample size for each tested recording device.
Data collection	Two sets of 61 pictures (Set A and Set B, N=122) representing natural and social scenes, humans, animals, objects and/or examples of abstract art were selected. The image database was deliberately varied in order to not preclude any exploration behavior by the tested populations. Each picture contained one or several salient features and was optimized for a 1280x1024 pixels screen resolution. The type and global content of the scenes were matched between the two subsets.<br>Each participant was instructed to freely explore each picture during a single session lasting approximately 30 minutes, one picture set using direct eye-tracking (≈15min), and a second picture set with digit-tracking (≈15min).
Timing	One single session lasting approximately 30 minutes for each participant.
Data exclusions	Due to a technical problem, data from two ASD patients during exploration of the set B images were not included in the analysis of eye-tracking data. (data corrupted and unreadable on the recording platform).
Non-participation	No participants dropped out/declined participation.
Randomization	Order of recording method and picture set (A or B) assignment were fully counterbalanced between subjects within each group.

Reporting for specific materials, systems and methods

We require information from authors about some types of materials, experimental systems and methods used in many studies. Here, indicate whether each material, system or method listed is relevant to your study. If you are not sure if a list item applies to your research, read the appropriate section before selecting a response.

Materials & experimental systems

n/a	Involved in the study	
☒	☐	Antibodies
☒	☐	Eukaryotic cell lines
☒	☐	Palaeontology
☐	☒	Animals and other organisms
☐	☒	Human research participants
☒	☐	Clinical data

Methods

n/a	Involved in the study	
☒	☐	ChIP-seq
☒	☐	Flow cytometry
☒	☐	MRI-based neuroimaging

Animals and other organisms

Policy information about studies involving animals; ARRIVE guidelines recommended for reporting animal research

Laboratory animals	Long tail macaque (Macaca fascicularis), 5yrs old, male

Wild animals	The study did not involve wild animals.
Field-collected samples	*For laboratory work with field-collected samples, describe all relevant parameters such as housing, maintenance, temperature, photoperiod and end-of-experiment protocol OR state that the study did not involve samples collected from the field.*
Ethics oversight	Tests conducted on non-human primates were in conformity with current guidelines and regulations on the care and use of laboratory animals (European Community Council Directive No. 86–609) and were conducted under a research protocol authorized by the French Ministry of Research Ethics board (APAFIS n° 2015061213048343).

Note that full information on the approval of the study protocol must also be provided in the manuscript.

Human research participants

Policy information about studies involving human research participants

Population characteristics	See above
Recruitment	Autism Spectrum Disorder participants : Representative patient population of the Azienda Ospedaleria Brotzu with clinical diagnosis of autism according the DSM-V. They were all young adults and rehabilitated patients.<br><br>Control population : Sex- and age- matched controls.
Ethics oversight	Tests conducted in human participants were approved by French (Sud-Ouest, project N° 2018-A02037-48) and Italian Ethical Committees (Azienda Ospedaliero-Universitaria of Cagliari, project N°AOB/2013/1, EudraCT code 2013-003067-59) and prior to the inclusion in the study, a written informed consent was obtained from all participants and/or their legal representative, as appropriate.

Note that full information on the approval of the study protocol must also be provided in the manuscript.
